# Women’s views on moderate and low alcohol consumption: stages of the subjective transition from pregnancy to postpartum

**DOI:** 10.1186/s12884-022-05247-0

**Published:** 2022-12-05

**Authors:** Jessica Pehlke-Milde, Irina Radu, Solène Gouilhers, Raphaël Hammer, Yvonne Meyer

**Affiliations:** 1 Research Institute of Midwifery, School of Health Sciences, Zurich University of Applied Sciences, Winterthur, Switzerland; 2 School of Health Sciences (Haute Ecole de Santé Vaud - HESAV), HES-SO University of Applied Sciences and Arts Western Switzerland, Lausanne, Switzerland

**Keywords:** Maternal health, Child health, Alcohol drinking, Pregnancy, Breast feeding, Qualitative research, Risk assessment, Consumer health information

## Abstract

**Background:**

Alcohol consumption during pregnancy and breastfeeding is associated with a risk for the child’s healthy development. Nevertheless, about 16 to 25% of all women in the European region, including Switzerland, consume alcohol during pregnancy and probably even more during breastfeeding. Little is known about how women perceive this risk and how risk perception changes during the transition to motherhood. The present study aims to explore the subjective transition from the woman’s perspective, focusing on perceptions of alcohol as a risk, changes in alcohol consumption in daily life and experienced support from health professionals in this period.

**Methods:**

The longitudinal qualitative, semi-structured interview study was jointly designed and conducted by health sociologists and midwifery researchers. Using the theoretical framework of sociocultural risk and life course transition, we interviewed 46 women from the French and German speaking part of Switzerland during pregnancy and until six months after birth.

**Results:**

In our study, we found that pregnant and breastfeeding women perceive alcohol consumption as a risk to the health of the child. Abstinence is sought especially during pregnancy, but this does not preclude occasional and low-level consumption according to some women. Alcohol consumption and risk perception change during the transition to motherhood. We identified five stages that characterise this transition in terms of alcohol consumption and risk perception. From the women’s perspective, there was a lack of counselling from health professionals, and the women expressed a desire for respectful and more individualised counselling.

**Conclusion:**

Many women express a need for guidance and counselling by health care professionals at some stages of the transition to motherhood. The stages identified can be used as pointers to address the subject of alcohol consumption in professional practice. The stage around conception and early pregnancy should be taken more into account, as women experience themselves as particularly vulnerable during this time. Low-threshold counselling services should be therefore offered to women before or in the stage around conception and be continued until the end of the breastfeeding period.

## Background

Stopping alcohol consumption is one of the key health recommendations for expectant and breastfeeding mothers in many western countries and most women cease alcohol consumption when they become pregnant [1]. Nevertheless, it is reported that about 10% of women worldwide consume alcohol during pregnancy and 16–25% of women in the European region, including Switzerland [2, 3]. During breastfeeding, results from international studies suggest that consumption is relatively common [4, 5]. Consequences of maternal alcoholism during pregnancy on the foetus’s development have been well-documented since the late 1950s and recognised in research and clinical practice since the 1970s [6]. Depending on the foetus’s degree of maturity, the amount of alcohol consumed and individual disposition, high quantities of alcohol consumed during pregnancy can lead to a severe physical and cognitive developmental disorder named Foetal Alcohol Syndrome (FAS), the most severe form of Foetal Alcohol Spectrum Disorders (FASD). Although the evidence is clear regarding high alcohol consumption and binge drinking and the severe consequences for child health [7, 8], the evidence regarding low to moderate alcohol consumption in pregnant women is heterogeneous and leads to different conclusions. Flak et al. [9], conducting a meta-analysis based on literature about the association between mild, moderate, and binge prenatal alcohol exposure and child neurodevelopment, conclude that there is no known safe amount of alcohol consumption during pregnancy. Another systematic review conducted by Mamluk et al. [10] focuses on studies of pregnant women estimating effects of low-to-moderate levels of alcohol consumption on pregnancy and longer term offspring outcomes. The authors highlight the paucity and poor quality of evidence and the limited evidence for a causal role of light drinking in pregnancy, compared with abstaining, on most of the outcomes examined. There is also limited evidence regarding the effects of low to moderate alcohol consumption by breastfeeding mothers on infant development. Some studies show that alcohol consumption may reduce lactation or disrupt infants’ sleeping patterns [11], but there is no clear impact on developmental outcomes [5, 12]. As there is no definitive boundary between harmful and harmless alcohol consumption during pregnancy and breastfeeding, and FASD is still a prevalent alcohol development disability for many children [13], public health recommendations in Western countries are based on the precautionary principle [14]. Pregnant and breastfeeding women are therefore advised to stop alcohol consumption no later than the onset of pregnancy to avoid any risk to the child. Generally speaking, pregnancy is perceived as an “at risk condition” [15–17] where medical risks need to be monitored by health professionals all through the pregnancy [18]. Health professionals offering antenatal and postnatal care are in a key position to ask women about their alcohol consumption and advise them that it is safest not to consume alcohol. In this rationale, it is the task of health professionals to increase women’s compliance to be abstinent, because alcohol is teratogenic [19], and the subject is too complex to be able to predict any safe threshold of alcohol consumption during pregnancy [20]. Other scholars question the general recommendation for zero tolerance in official guidelines, especially due to the lack of evidence regarding the effects of light alcohol consumption [10, 21]. Mamluk et al. [10] conclude that the unclear distinction between light drinking and abstinence is the biggest challenge for health professionals and pregnant women, leading to inconsistent recommendations and advice. Indeed, several studies indicate that health professionals give discordant advice to pregnant and breastfeeding women, from strict abstinence to tolerating low occasional consumption [22, 23]. Many authors therefore recommend consistent information about the effects of alcohol consumption on the developing baby as well as education for health care professionals to improve their counselling skills [14, 24–27]. Information and counselling about alcohol consumption seems to be widely accepted by pregnant and non-pregnant women [28] and there is a clear need to address this topic in routine antenatal and postnatal care [24]. Additionally, a high amount of responsibility is attributed to the women themselves, who in public discourse are seen as mainly responsible for the foetus’s wellbeing and thus for risk minimisation [29]. As alcohol consumption during pregnancy and breastfeeding is associated with taboos and fear of stigmatisation, a conversational approach based on a trusting relationship seems promising to increase women’s readiness to talk about their habits [30]. Furthermore, understanding the individual perspective of women and their social context is necessary for the design and successful implementation of women-centred health interventions [26].

This is the reason why women’s perception of risk is at the centre of our research. Our approach to the concept of risk is sociocultural, which means that variations in individuals’ conceptualisations of and responses to risks are not only prompted by intrinsic characteristics of the danger itself but are shaped by the particular sociocultural settings in which individuals live [31, 32]. Within this theoretical paradigm, we adopt a weak-constructionist approach, which does not deny the existence of objective risk, but posits that risks are culturally biased based on societal framing and personal experience. Risks are also viewed as mobile and not static, but as changing over time in response to new experiences and new information [33]. In connection with alcohol risk, previous research from French-speaking Switzerland has shown that alcohol consumption guidelines are contextualised and interpreted by pregnant women, and that occasional alcohol consumption during pregnancy can sometimes be deemed acceptable and be perceived as comparatively less risky than smoking [34, 35]. Despite this, abstinence or strong alcohol reduction are largely envisioned by women as ideal strategies of dealing with alcohol risk during pregnancy, with feelings of guilt and anxiety over alcohol consumption during pregnancy often being concomitant to these exceptions [36]. While other longitudinal qualitative studies analysing the development of health risk perception over time exist [37, 38], to our knowledge, there are no studies that have examined women’s perceptions of the risk of alcohol consumption during the transition from pregnancy to breastfeeding. To frame this approach, we refer to Levy [39], who defines the concept of “transition” as a period of change in the individual life course perspective. If in this paradigm life stages are considered as relatively stable states in the life course of an individual, then transitions mark the periods of change between stages, periods in which development takes place [40]. Taking the theoretical perspectives of sociocultural risk and life course transition into account, the present study aims to explore the subjective transition in its entirety from the woman’s perspective, focusing on perceptions of alcohol as a risk, changes in alcohol consumption in daily life and experienced support from health professionals in this life span.

## Methods

This article is based on data from a study in which we conducted interviews with 46 heterosexual couples in Switzerland with the aim to understand how they perceive and experience alcohol consumption during the transition to parenthood. In keeping with the longitudinal approach, couples were interviewed at two points in time: first during pregnancy, between 20 and 40 weeks, when both partners were interviewed separately (thereafter referred to as “pregnancy interviews”) and second at between 12 and 24 weeks postpartum, when only the women were interviewed (hereafter referred to as “breastfeeding interviews*”).* For pragmatic reasons, we decided not to interview male partners twice, since in a previous pilot study their recruitment was more difficult overall, but especially in the postpartum phase. The whole corpus includes a total of 138 interviews. Since this article focuses on the transition to motherhood, this paper is based on the 92 interviews conducted with the women (46 pregnancy and 46 breastfeeding interviews). The interviews took place half in the German- and half in the French- speaking regions and were conducted in Swiss German, German, French and English. The longitudinal qualitative, semi-structured interview study was jointly designed and conducted by health sociologists (RH, IR, SG) and midwifery researchers (YM, JPM). Thus, the study combined knowledge about how sociocultural settings may shape individuals risk perceptions as well as that of perinatal health and maternity care in both language regions. We conducted all phases of research in accordance with the ethical guidelines of the Swiss Academy of Humanities and Social Sciences, which follow the principles of the Declaration of Helsinki. The study protocol was submitted to the Swiss Association of Research Ethics Committees, who ruled that our study did not fall within the formal scope of the Swiss Federal Act on Research involving Human Beings [41].

### Data collection

Couples were eligible to participate in the study if the woman was not abstinent from drinking alcohol before she got pregnant, if she was not treated for alcohol addiction before or during pregnancy, and if she intended to breastfeed. These exclusion criteria were set because we were interested in couples with average alcohol consumption patterns and the way they reduced and changed their alcohol use. We recruited couples in French-speaking and German-speaking areas of Switzerland using various channels, such as displaying flyers or presenting the study in maternity hospitals and birth centres as well as in antenatal classes and prenatal yoga classes. Recruitment proved more difficult than expected and support from stakeholders was limited due to the perception of the study topic as being sensitive. As interviewing couples requires both individuals to give consent to participate, access was prioritised through female participants. Whenever possible, women were first asked about their willingness to participate in the research. If they were interested, they were asked to provide the researcher with their partner’s contact details. However, in situations such as antenatal classes where both partners were present, they were both asked about their interest in participating in the study. Prior to the interviews, both partners were sent a written information sheet describing the study and the consent form, which they signed in the presence of the interviewer after all questions had been clarified. All interviews were carried out face to- face except for four interviews with women and five interviews with male partners, which were done by Skype or telephone in order to accommodate the priorities of the participants. Interviews were conducted in French, German and Swiss German, and a few in English, by two researchers experienced in qualitative interviewing (SG and IR). With one exception, each couple was interviewed by the same interviewer. The “pregnancy interviews”, conducted from October 2017 to August 2018, lasted between 60 and 80 min. The “breastfeeding interviews” lasted on average 60 min and took place from March 2018 to March 2019. All interviews were conducted at a location of participants’ choice and were digitally recorded and transcribed verbatim. All participants’ names were replaced with pseudonyms and identifying features were removed from the transcripts. Participants’ quotes used in this paper were translated from French or German.

### Data analysis

Prior to the interviews, we developed semi-structured interview guides based on the theoretical framework and previous research. The pregnancy interview guide for women covered the following topics: experience of pregnancy and antenatal care, changes in daily life habits, alcohol consumption before pregnancy, changes in alcohol consumption and perception of consumption as a health risk. Pregnancy interviews focused on participants’ personal experience while also addressing their view of their partner’s experience and their functioning as a couple during pregnancy and their plans after the birth. The breastfeeding interview guide addressed women’s experience of post-partum and breastfeeding, experience of social support or lack thereof, including changes in everyday life since the child’s birth, changes in alcohol consumption and the perception of alcohol as risk. We analysed the data drawing on the principles of longitudinal qualitative research as a flexible methodology combining deductive and inductive strategies [42, 43]. We created a summary document for each of the 46 couples in order to preserve the contextual meaning of their accounts, as well as the longitudinal development of their experiences and perceptions with regard to the analysis themes. This served as a basis for the subsequent longitudinal analysis. For this article, we additionally compared the women’s interviews with each other and identified transversal themes that marked a transition. Throughout the course of the project, the research team met regularly to discuss the emerging findings and their interpretations, enhancing the trustworthiness of data analysis [44, 45]. All interviews were analysed using software Atlas.ti.

## Results

No participants withdrew from the study. In our sample most women were expecting their first child (*n* = 27), 13 had one child and 6 had two children or more. Most women breastfed exclusively or partially for a minimum of three months, except four women who stopped breastfeeding early in the postpartum phase. The women were aged between 27 and 43. Most participants spoke Swiss German or French as a native language, some spoke German (*n* = 12) or a different native language (*n* = 11). The participants overwhelmingly had a tertiary level of education (39 out of 46), and a certain number of women working in health care (nurses, midwives, healthcare technicians and doctors) were present in our sample (16 out of 46). Among our participants the majority were cared for by ob-gyns during pregnancy. In the following description of our results, we will refer to pregnancy interviews as “P” and breastfeeding interviews as “BF”.

In our interviews, alcohol consumption and the way it is perceived as risk by the women changes during the transition to motherhood. We identified five significant stages in the subjective transition regarding the perception of low and moderate alcohol consumption as risk: (1) Around conception and getting pregnant: the intangible risk of alcohol consumption, (2) Manifestation of pregnancy: weighing the “psychosocial” and “medical risk” of alcohol consumption, (3) Being pregnant: dealing with the concept of abstinence, (4) The first weeks after birth: alcohol consumption is incompatible with childcare and (5) The public mother: the risk of being criticised for consuming alcohol.

### Around conception and getting pregnant: the intangible risk of alcohol consumption

In our interviews, women describe the stage of the expected but not yet confirmed pregnancy as a challenging one in terms of assessing the risk of alcohol consumption. As the pregnancy is not yet ascertainable, the risk for the embryo also remains intangible. Rahel, who is expecting her first child, qualifies this early phase of uncertainty as a difficult one, when around *“day 25 of the cycle”* you might be pregnant, but you don’t actually know, if you are. During this time Rahel did not stop alcohol consumption and was unsure if she should. Stefanie, who suffered several miscarriages before her current pregnancy, also did not stop drinking before her positive test, explaining that she *“didn’t want to punish herself”*, by being abstinent in view of the insecure nature of the pregnancy at this early stage. Women, like Anna, who stopped drinking altogether before pregnancy to prepare the body for pregnancy, were in the minority in our sample. However, many women had already begun to adapt their alcohol consumption to varying degrees, for example by avoiding binge-drinking or reducing the frequency of their consumption. Other women, like Mia, who was now expecting her first child, drank only beverages with a low percentage of alcohol, perceiving them as being less dangerous for the embryo:

*“Yes, I mean, I’m already someone who doesn’t drink too much alcohol. I won’t have a glass of wine with each meal. But sure, I also liked going to an apéro or something. I did reduce my alcohol consumption, because I felt like, if it does happen, I don’t want to have a guilty conscience, because I had alcohol. So, I was careful with what I drank, I didn’t drink any hard liquor, but a glass of wine or things that don’t have a high percentage (of alcohol).“*(Mia, P).

Most women in our sample were in long-term partnerships and recounted that the pregnancies were planned or at least desired. Therefore, most women suspected early on that they might be pregnant, except for one woman who reported the pregnancy later and consumed alcohol during that time. The confirmation through the pregnancy test was usually an important step, as it was the moment when most women stopped drinking alcohol, some describing a sudden lack of interest in it. Almost as an instantaneous effect Stefanie and Paulina described being “*done*” with drinking alcohol, the moment they got the confirmation that they were pregnant.

As women perceived the confirmation of the pregnancy through a positive pregnancy test as an important moment, one where they were expected to immediately start changing their dietary and alcohol consumption habits, some women purposefully delayed taking the test. Marie for instance describes delaying her test to one week after a particular weekend, when she knew she would be “*drinking, smoking, eating tartars and salads*”, habits which she perceived as being incompatible with pregnancy, although she did not really consider them dangerous for the embryo at the beginning of the pregnancy. Thus, delaying the pregnancy test meant delaying the official beginning of the pregnancy and the responsibility and social expectations that come with this state.

How much women had to reduce their alcohol consumption at the beginning of their pregnancies depended a lot on their age, professional context and family situation. Some women, like many of the ones who were already mothers, had a very low alcohol consumption, because of long-term breastfeeding, or their overall demanding family life. For these women, alterations of their alcohol consumption were hardly necessary. For many of the first-time mothers, however, as well as for women whose work or private life involved regular socialising accompanied by alcoholic beverages, the adaptation was more constraining. Isalyn, expecting her third child, explains the difference between her first and second pregnancy in terms of changing her habits of alcohol consumption:

*“The first time, what was striking was the fact that I didn’t drink alcohol anymore, that I didn’t go to aperitifs with our friends, in fact one felt completely out of step (…). So, the second time I experienced it differently because I had already mourned what I couldn’t do and then there is the rhythm of parenting, which is already in place.”* (Isalyn, P).

For Marie, just as for Mia, the early stage of the transition to motherhood is accompanied by an emerging feeling of responsibility for the child’s wellbeing and guilt, which are dealt with in different ways. For some women this responsibility can be stressful, especially for those who consumed alcohol when they did not yet know they were pregnant and who deem the consumption potentially dangerous for the child. Patricia for example recalls how, during her first pregnancy, she considered an abortion because she feared that her alcohol consumption early in the pregnancy, might have harmed the foetus. She describes contacting a local association against alcoholism, in order to discuss her insecurities, which then tells her to “*relax*” and not be too troubled by this early consumption, as at this point in the pregnancy, according to the association, it’s the “*all or nothing principle*”. Aside from this example, in this very early stage of pregnancy, most women report that discussions with health professionals are largely absent, or only occur once the pregnancy is confirmed by a doctor.

### Manifestation of pregnancy: weighing the “psychosocial” and “medical risk” of alcohol consumption

After the pregnancy is confirmed, there is a stage when several of the women we interviewed did not want to disclose the pregnancy to their wider social circle because they feared complications such as a miscarriage in the first three months. During this phase women often described the pregnancy as externally invisible, as they often did not yet physically appear pregnant. Abstinence, in this context, was perceived by women as an outwardly recognisable manifestation of pregnancy, which risked breaking the confidentiality of the pregnancy, as Jana recounts.

*“I was at the Christmas party and usually I like to drink some alcohol and it became really apparent, that I didn’t drink alcohol, and it really annoyed me; I can understand why someone might make a quick remark: aha you’re not drinking alcohol, are you pregnant? But it’s so intrusive.”* (Jana, P).

At this stage, disclosing the pregnancy too early may be perceived as a psychosocial risk, which from the women’s point of view must be weighed against the medical risk of alcohol consumption. Indeed, some women weighed the potential consequences of a small consumption of alcohol at this early stage of pregnancy against the possible consequences of disclosing the pregnancy too early and found the secrecy of the pregnancy to be more important. Amanda describes having to *“pretend she drank”* and also drinking *“one or two glasses”* until she was twelve weeks pregnant, even in front of her parents, who did not know she was pregnant. In retrospect, she describes how this prioritisation changes during the course of pregnancy.

*“At the beginning of the pregnancy, the priority is that it’s a secret and then later on in the pregnancy, the priority is the baby. (…). It’s a bit strange that we sacrifice the safety of the baby at that moment, even if the sacrifice is probably minimal, but it’s strange that we sacrifice the safety of the baby at the beginning of the pregnancy, just during embryogenesis, when it’s most crucial, because it’s so important that it’s not known.”* (Amanda, P).

Amanda’s passage reveals the dilemma in which many women find themselves. On the one hand, there is the expectation of being a good mother who protects her foetus from possible harm and is therefore abstinent. On the other hand, the interviews reflect the social expectation that a woman should keep her early pregnancy private, until the risk of miscarriage is reduced.

This stage of transition coincides with the period when women report having attended at least one antenatal check-up. While most women report having received dietary advice at this check-up, only a minority talk about alcohol consumption with their doctors. Lena, who is expecting her second child, explained that she does not dare to address the issue directly:

*“Have you ever discussed this with the gynaecologist?“*

*“No.“*

*“She didn’t bring it up?“*

*“No.“*

*“And you didn’t ask it either?“*

*“No. Because it just makes me feel bad about asking how much alcohol I can drink. I think it already has a stigma.“* (Lena, P).

Only a minority of the women in our sample recalled that the issue of alcohol consumption had been addressed by health care professionals during these initial check-ups. Most reported that they were not advised in detail, but that a recommendation was given, which sometimes was not compatible with their personal views. Barbara, for example, who continued drinking some wine occasionally during the first few months, as it made keeping the pregnancy private easier, discussed this early consumption with her gynaecologist and received the answer that *“pregnancy means zero alcohol*”. This left her feeling unsure about whether she had put her child at risk. Other women, however, like Cornelia, received an entirely different advice, being told that a small occasional consumption of alcohol is not perilous for the foetus. She describes how she knew that she does not want to drink any alcohol during her pregnancy, despite different advice from her doctor:

*“I’ve heard from different sides, even from my doctor who told me that I could have a glass of Prosecco (sparkling wine) on Valentine’s Day. But I would have had a much too guilty conscience and even one sip wouldn’t have given me any pleasure.”* (Cornelia, P).

Although the tension between the perceived risk of alcohol consumption and the advice of health professionals was perceived as stressful, this issue was not addressed further, neither from the women’s nor from the health care professionals’ side. As a result, the women in our sample were largely left to their own devices with the challenges of assessing and weighing the risks.

### Being pregnant: dealing with the concept of abstinence

As soon as the pregnancy was announced or visible, women described that abstinence was more easily accepted, endorsed, or sometimes even demanded by their social circles. However, some women explained that the opposite could also be true: people in their families, circle of friends and occasionally even health professionals, encouraged occasional low consumption at special events. These women were often confronted with the expectation that they should be more relaxed and not enforce health recommendations too drastically. Some women, like Anna, were faced with both expectations.

*“It’s funny, you can see the way people look at you. You have the ones who say, “go on, one glass isn’t going to harm you”. That’s especially the older generation, it’s quite funny (…) and then younger people say “oh, you’re drinking?”* (Anna, P).

Being abstinent at this stage of pregnancy seemed to most of the women interviewed to be a benchmark for the role of a good, caring mother who takes her responsibility towards the child seriously. A few of the women even saw this as an element of control that they had over their pregnancy and their baby’s health. Denise for instance, who was expecting her first child, explains that *“It (abstinence) wasn’t that hard because I know exactly why I’m doing it and it’s not forever”*, while Stefanie says that *“it’s (abstinence) something I can control, so I do”.*

The experience of changing from non-abstinence to abstinence is sometimes seen in relation to pre-conceptual alcohol consumption. For Patricia, who talks about practices of alcohol consumption in her family and thinks that she and her partner drank too much in the last two years, “*giving up alcohol still changes a lot*”. For her and her partner, pregnancy was also an opportunity to rethink and change their consumption habits: *“He drinks less since we stopped drinking together”*. Hélène, on the other hand, explains that her pre-conceptual alcohol consumption was minimal because she does not like wine. For her, the transition to abstinence was *“not a big sacrifice”.*

However, many women were conflicted about the actual definition of abstinence simultaneously referring to themselves as being abstinent, while ruefully admitting that there had been small, occasional exceptions, which they didn’t know how to classify. Thus, there was uncertainty among the women who wanted to be completely abstinent about how to interpret this term; does abstinence also mean not cooking with alcohol, is non-alcoholic beer allowed, is it okay to take a sip from your partner’s glass at a social gathering? Women’s practices around these uncertainties varied. Some, like Stefanie, decided to cook without alcohol and send back dishes in restaurants that seemed to contain alcohol. Unfortunately, there were limits to what they could influence, as Lena found out when she thought she was being safe by drinking non-alcoholic beer during her second pregnancy, only to later realise that it also contained a small percentage of alcohol, a realisation that “*scared*” her. Similarly, Stefanie, took medication which contained alcohol and was similarly worried once she realised it afterwards.

Conversely, what was an exception from the no alcohol rule they had imposed on themselves, was also a question. For some women, having “*a glass of wine at Christmas with my meal*” (Isalyn, P), or “*having a glass with a meal every two weeks*” (Anja, P) might be acceptable, in terms of risk for the child’s health. At the same time for many of them, exceptions, however small, are also linked to a lot of guilt and doubt:

*“I may have had one every 2–3 months, when there’s a bottle of champagne and I’ll have a quarter glass, but I feel guilty about it. As soon as I have two sips of alcohol, I tell myself that it’s terrible for the baby, and that I shouldn’t.“* (Lou, P).

Many of our interviewees explained that as time went by and the pregnancy progressed, they started feeling left out during social gatherings, feeling an increased sense of separateness and distance from their social circle. Some reported a more direct exclusion, such as friends meeting without them and referring to them as *“no longer fun”* now that they are pregnant and cannot drink (Aurelia, P). For most women, this was a more implicit gradual process, in which they started feeling more and more *“different”* from their circles. Some felt like their habits as pregnant women, who need to leave out certain dietary ingredients and not drink alcohol, is somehow being *“complicated”* or *“difficult”*, which makes them feel *“annoying”* (Stefanie, P).

Similarly, some of the women reported feeling that a quality of comfort and relaxation was missing from their social interactions in the absence of alcohol consumption. For some, this was seen as a temporary inconvenience that was readily accepted because of the time limit. For other women, however, this same feeling plays a part in consciously allowing themselves a low consumption of alcohol during pregnancy. Dalila, for example, is well informed about the studies on alcohol consumption during pregnancy, and she believes low consumption to be tolerable during pregnancy. She appreciates the feeling of relaxation this low consumption allows her in social situations:

*“I was relatively far along (in the pregnancy) over Christmas. I think eating well and drinking wine are valuable things. And then just doing without or just taking two or three sips is just not the same (…). Now it’s spring and if you go out and have a sip of beer, I don’t think that’s so bad.”* (Dalila, P).

Like Dalila, Rahel is a woman who cultivated an alcohol consumption during pregnancy, which she describes as *“half a glass of wine*” at social events. She qualifies this consumption consciously as *“more than an exception”* and as *“chosen moments”.* She explains that while the component of relaxation also plays a part, another part of her motivation is the conviction that the dietary and alcohol consumption guidelines for pregnant women are exaggerated:

*“I’ve already been interested in the guidelines and the criticism of the guidelines for my academic work. And then what I say to myself is, that first of all, we don’t know. If they really knew that half a glass of wine every fortnight is very, very serious for the baby, we would know that. (…). So, I’m maybe a little bit in reaction against the kind of posture that is a little bit too bossy, especially coming from North America where the philosophy is completely different and the list of things you shouldn’t eat is quite long.”* (Rahel, P).

All women in our sample reduced their alcohol consumption significantly in comparison to their previous alcohol consumption habits. In general, alcohol consumption during this stage of transition was partly discussed with the social environment, especially with friends and family, as well as the partners, who were sometimes consulted when the women were unsure what to do. According to the women’s accounts, these doubts about instances of small consumptions and exceptions from abstinence were rarely discussed with health care professionals. Some women who had the necessary health literacy specifically researched study results on alcohol consumption during pregnancy.

### The first weeks after birth: alcohol consumption is incompatible with childcare

Most of the women in our sample reported that they had read or heard that some alcohol consumption during breastfeeding can be safe if the interval between consumption and breastfeeding is long enough. According to the women, this interval could only be maintained after a breastfeeding rhythm had established or, for some, when solid food was introduced. Drinking alcohol before this moment when feedings are sufficiently far apart is described by many women in our sample as logistically difficult and stressful:

*“So still no alcohol. I mean, sure, you can drink alcohol, pump it out. It’s just too stressful for me, I have to be honest.”* (Mia, BF).

Other women, like Ronja, describe having to count the hours between consumption and feedings as simply “*not worth it*”. On a broader scale, describing the context during the first weeks after the birth, many women explain that their birth experience, for some including caesarean sections, long labour and other unexpected complications, left them especially tired. Some women like Jana, who suffered from post-partum insomnia, feared consuming alcohol would make her even more tired. Others, like Lena, who had an unplanned caesarean section that left her feeling guilty and worried for her baby’s wellbeing, was simply too preoccupied during this early period to contemplate drinking. Most women, however, simply described being too overwhelmed with the responsibilities of childcare and managing breastfeeding. Manuela, who has a young child and now the baby, said that “*drinking alcohol is out of the question*” in a context where she barely has time to shower during the first months after the baby’s birth. Aside from the issue of physical tiredness, women highlighted the issue of responsibility. As Ramona explains, the desire to drink alcohol vanished for her during this period, with her focus being on her maternal responsibility:

*“Yes, but you don’t feel the desire. Because you have the feeling that you have to be there for your child. And somehow the care doesn’t stop (laughs).”* (Ramona, BF).

Other women, like Yvana, explained that, in the context of the great tiredness she experienced from breastfeeding, alcohol consumption was perceived as more of a stress, than a relaxation, and an impediment to being able to fully manage the responsibilities of breastfeeding and childcare:

*“It’s also something, between the tiredness, the tiredness of breastfeeding as well, I don’t react the same way to alcohol. And I feel that I won’t be able to drink again (…) if I drank in the evening, if we celebrated something, I wouldn’t be able to manage the night of breastfeeding afterwards, if she woke up, that would be sure.”* (Yvana, BF).

Although all these women have male partners who are involved in childcare to different degrees, the responsibility for the baby’s wellbeing during this early stage of motherhood is experienced by most women as being primarily in their hands. As the main caregiver during the first months of the baby’s life, the pressure to perform this task as well as possible is thus very high. Another factor influencing non-alcohol consumption during this stage of the transition to motherhood is the relative isolation that some new mothers find themselves in. Jana, for instance, describes how she would have barely had any occasion to drink, had she wanted to, as she was mostly at home alone with the baby:

*“Yes, I never went out because it’s so hard to sleep, so I never went out to eat in the evening or anything like that. There were also no social situations where I could have had a drink.”* (Jana, BF).

At this early stage, discussions with health professionals rarely touched upon the issue of alcohol consumption. Most of the women who sought advice were dealing with other issues, such as breastfeeding problems and rhythm, how to sleep through the night, and how to deal with other physical problems such as pain. Alcohol consumption would thus return to the women’s focus only in the next stage of the transition to motherhood, as breastfeeding frequency would decrease and a return to more sociability and activities that are independent from the baby would begin again.

### The public mother: the risk of being criticised for consuming alcohol

After a first period with the baby and the establishment of a breastfeeding rhythm, life normalised again somewhat and some women started occasionally drinking alcohol and some women started going out a little more. However, many found that the judgment of their social circles on their alcohol consumption, was a frequent companion.

Ygritte, despite her efforts to breastfeed, had to stop around the third week after birth. She then fed her baby formula milk. She described how going into the restaurant with the baby, she often felt judged by people around her, when ordering a glass of wine. Other women, like Dalila, describe how she found herself being questioned by friends and family after her first pregnancy, when she wanted to consume alcohol while breastfeeding, despite being a health professional herself. She would then explain her standpoint, something which she stopped doing after her second pregnancy:

*“With the first child I still discussed and explained my point of view, now I think, no. I no longer have to justify myself to everyone. It’s enough for me to say that I made this decision, and I can stand behind it.”* (Dalila, BF).

Some breastfeeding women report that friends or relatives encouraged them to drink some alcohol. If the women regarded the people encouraging them as trustworthy, alcohol consumption may sometimes be perceived as safe. Lena, who did not discuss alcohol consumption during breastfeeding with her midwife, describes an interaction with her brother-in-law who is a physician and a father as well:

*“And we were there on holiday, and he did a barbecue and he said do you take a little wine as well? Then I said no. And then he said it doesn’t matter in the case. Then I said, all right. And this gave me like a little bit of safety because he also has two children.”* (Lena, BF).

As during the pregnancy, health professionals generally did not discuss the topic of alcohol consumption during breastfeeding and women themselves did not ask, this even though other dietary restrictions were discussed.

The motivation for breastfeeding for most of the women was deeply linked with the baby’s wellbeing. Women perceived breastmilk to be the best kind of nutrition they could offer the baby in terms of protection against allergies and overall immunity, as well as in terms of bonding. Therefore, most were motivated to breastfeed for as long as possible. For some, like Dalila or Paulina, the endpoint of breastfeeding needed to be adapted to the baby’s needs, while for others, like Lena, keeping up with the guidelines recommending six months of breastfeeding was particularly important. None of the women mentioned wanting to take up more regular alcohol consumption as a reason for shortening breastfeeding. While stopping breastfeeding was interpreted by the women as a moment that would free them of the obligation to curtail their alcohol consumption some women looked forward to, many described the transition to motherhood as a time that radically changed their habits and priorities.

Indeed, talking about how the women viewed their alcohol consumptions in the future, many mentioned that they were not sure that they would ever return to their pre-pregnancy alcohol consumption habits. Vanessa describes the difficulty of being able to go out, on the one hand, as a mother of three, which already limits the occasions where she would consume alcohol, as well as the sensation that something has changed in the way she metabolises alcohol after giving birth to three children. She explains that she ultimately does not want to return to her prior alcohol consumption habits:

*“No, not really. I just realise that I don’t really metabolise it well anymore. Maybe everyone says that (laughs). If I have a glass of wine with dinner, by the time I drank half of it, I already feel tipsy. Before I could really have a few glasses. But it’s not that bad.”* (Vanessa, BF).

Here we again notice a difference between first time mothers and women who already have children, with the former being more inclined to want to take-up a similar alcohol consumption as the one before the pregnancy. In retrospect, the women we interviewed regretted that they had received so little support from health professionals on how to integrate alcohol consumption in a responsible and safe way during pregnancy and breastfeeding.

## Discussion

In this paper we examined women’s change in their perception of risk of alcohol consumption, as well as their experience of consumption. Furthermore, we analysed the support they received from midwives and doctors during the transition to motherhood, meaning the time before conception, the pregnancy itself and the first six months after birth. Using the theoretical framework of sociocultural risk and life course transition we identified five stages which mark the transition regarding low and moderate alcohol consumption from the point of view of the interviewed pregnant and breastfeeding mothers.

### Changing alcohol consumption in daily life

Our findings show that alcohol consumption changes during the transition from pregnancy to breastfeeding, with the identified stages characterised by periods of change. In the first stage, when pregnancy is not yet confirmed, most of the women we interviewed consumed alcohol, but avoided drinking large amounts or strong alcohol. Only a few women had stopped drinking altogether before pregnancy. This result has been reported before and suggests that alcohol consumption is higher in this stage than after pregnancy is confirmed [1]. In the second stage, when the pregnancy is recognised but not yet publicly known, all women reduce their alcohol consumption significantly, but some consume very small amounts of alcohol in certain situations in order not to reveal the secret of the pregnancy [38, 46, 47]. If the pregnancy is known, women develop different strategies around abstinence during the third stage, only few of them allowing themselves some small amounts of alcohol linked to special events. Shortly after birth, in the fourth stage, alcohol consumption is not an issue for the women interviewed, as they are challenged by caring for the new-born [46]. In the last fifth phase after birth, particularly first-time mothers describe a reduction of alcohol consumption compared to the time before pregnancy.

The change in alcohol consumption is particularly pronounced in early pregnancy, when women change their usual drinking habits to the extent that they completely stop or drastically reduce their alcohol consumption. The change is also clear up to six months after birth, when women slowly resume alcohol consumption. This finding is consistent with results from previous studies indicating this marked adjustment around early pregnancy, as well as that alcohol consumption increases again after the birth of the child [1, 4, 48]. In particular, first-time mothers describe that alcohol consumption before getting pregnant is closely linked to certain social activities, having a glass of wine with friends. For some women abstaining from alcohol is therefore accompanied by a reduction in social participation and women experience the state of being excluded or different as part of their transition to motherhood. The withdrawal from social life also applies to some extent to the postnatal period, which leads to a lower alcohol consumption [49]. Here, the women in our sample not only experience a normatively based ostracism of the “drinking mother” [46] but also describe a certain physical intolerance that is connected with alcohol consumption. Some women who are already caring for small children report that their alcohol consumption is already reduced significantly.

### Perception of risk of alcohol consumption

In our study, we found that women perceive alcohol consumption as a risk to the health of the child, especially during pregnancy, and therefore drastically reduce or stop alcohol consumption to minimise the risk. Most of the women in our corpus were aware of the official recommendations of abstinence during pregnancy and most reported following them as closely as possible, some of them without exception, to control the risk as much as possible. These women were sometimes irritated when they were confronted with a relativisation of the risk by midwives and doctors from their point of view. Others, especially multiparous and postpartum women, took the position that controlled and infrequent alcohol consumption was compatible with their perception of risk and allowed themselves some consumption. Lastly it must be mentioned that in our corpus, the definition of abstinence was relative. Consequently, what constituted an exception to abstinence varied from woman to woman and was shaped by the individual’s perception of risk, as well as on their social and family context.

Some women who did not know they were pregnant and had consumed alcohol in early pregnancy reported long-lasting strong feelings of guilt and worry that they had seriously harmed the child [15]. After the pregnancy was confirmed, some women occasionally consumed small amounts of alcohol. This controlled consumption of wine or beer during pregnancy was usually not considered as a serious health risk to the child, but most of the women interviewed experienced a degree of insecurity that worried them. This uncertainty about the residual risk described by women reflects the lack of sound scientific evidence regarding the topic of low alcohol consumption during pregnancy [10]. Women were usually unsure about the extent of the risk, even while breastfeeding, and lacked expert advice from midwives and doctors [24, 26].

There were also women who were aware of the existing studies on the uncertainty of the effect of small consumption taking the position that occasional low consumption is generally compatible with responsible motherhood. Some of them were generally critical of the many guidelines pregnant and breastfeeding mothers are supposed to follow today (alcohol, diet, sport), arguing that the abundance of guidelines and the associated control over women’s bodies place a great burden on women and lead to over-responsibility and stress during motherhood. Despite this critical attitude, some of these women avoided any or limited their alcohol consumption to prevent any risk for their child. They explain that they acquiesce to the pressure of the societal risk discourse in female reproduction because of their sense of responsibility towards their unborn child, but also because of the fear of being held responsible by others [17, 29]. Despite these variations, all interviewed women related their perception of risk to the concept of “good motherhood”: thus working hard on their identity as good mothers meant being risk-averse [28, 50]. First-time mothers in our sample tended to differ from second or multiple mothers who had already gained experience and were able to draw on this in subsequent pregnancies. This also meant that the perception of risk differs from pregnancy to pregnancy, and alcohol consumption changed, for example in that abstinence was handled more flexibly or more rigidly [36].

### Experienced support from midwives and doctors

Modern concepts of maternity care emphasise approaches which empower women in informed decision making and strengthen their health literacy, so they can understand and apply information that impacts their and their children’s health [51]. In this study, women across all stages consistently reported that professional advice on alcohol consumption during pregnancy and breastfeeding was either completely absent or inadequate. When professionals provided information on alcohol consumption, such as in the context of pregnancy confirmation, it appeared to be general and not tailored to the individual needs of the woman. From the point of view of the women interviewed, it was difficult for them to address the taboo subject on their own initiative and to disclose the need for counselling [52]. The minimum requirements for making a well-informed decision about alcohol consumption during pregnancy and breastfeeding were therefore not met from the women’s point of view. Results of this study thus confirm lacks already identified in other contexts about professional information and counselling on alcohol consumption during pregnancy and lactation [22, 53].

Women in this study seemed to be informed about the general recommendation for abstinence during pregnancy and breastfeeding. Nevertheless, the results show that especially among first-time mothers around the passage of conception and early pregnancy, a strategy of how to deal with alcohol consumption is not yet consolidated. Some women report feeling vulnerable in this stage, when they sometimes consume small amounts of alcohol during social events [19], in order not to disclose their pregnancy. In a cultural context, where announcing pregnancy too early is not the social norm and abstinence is considered an indicator of pregnancy, women face the conflict of balancing social and medical risk. Since, from the women’s point of view, a gap in care is shown here, professional support for women in the phase around conception should be reconsidered [22, 28]. However, results of this study indicate that from the women’s perspective, there is no need to improve the health status of women (e.g. by reducing alcohol consumption) before conception through public health interventions, as suggested by many studies [54]. In fact, the need could rather be seen in discussing the taboo topic of alcohol consumption in an easily accessible and protected setting and in clarifying how women can address challenging situations [55].

Official guidelines emphasise the importance of early assessment of alcohol consumption mostly focusing on identifying women at increased risk and transfer to other health care specialists. In fact, late detection of pregnancy seems to be one of the strongest maternal predictors of foetal alcohol spectrum disorders [56]. Results of this study suggest that women who in principle want to follow the recommendation for abstinence are not being reached specifically enough and are left to their own devices. This does not only apply to the early phase of pregnancy, as the results suggest that there is a differentiated need for information and respectful and non-judgmental counselling throughout the transition to motherhood. Health care professionals and doctors should therefore be informed that a single consultation at the beginning of pregnancy does not cover the changing needs of women during pregnancy and breastfeeding [25]. Rather, the stages identified in this study could give an indication of the needs at different stages. Midwives who provide continuity of care therefore seem particularly encouraged to meet individual needs for support during pregnancy, birth and breastfeeding, and to strengthen women’s capabilities in the context of respectful relationships [57].

During the transition to motherhood, advice from the social environment can be experienced as supportive, especially if it comes from trusted persons from the immediate social environment. However, comments from the social environment can also be experienced as very distressing. Both pregnant and breastfeeding women seem to be the subject of public judgement and their behaviour regarding alcohol consumption is critically commented on, regardless of whether the woman is abstinent or not. Views on alcohol consumption and corresponding advice are heterogeneous and it is therefore impossible for women to please everyone. Counselling approaches that address such critical situations and involve the woman and her inner social circle could contribute to empowering women [53, 58, 59].

### Limitations

Most of the women in our sample had tertiary education and were thus of a high socioeconomic background and lived in stable partnerships. Many of them have the health literacy to inform themselves about the effects of alcohol consumption and to critically reflect on studies. The study population is therefore, at least partially, informed about the lack of scientific evidence regarding low alcohol consumption during pregnancy and breastfeeding and may be critical of generalised health messages. Further studies with a more diverse sample in terms of socioeconomic background, sexual orientation and family situation would be needed. Another limitation is that the study did not aim to measure alcohol consumption. Indeed, as a qualitative study, the aim was for consumption habits and attitudes toward alcohol to be depicted in depth by the women, in their own words. Although most of the consumption habits related by our interviewees can be described as occasional, they cannot be equated with “low and moderate alcohol consumption” as corresponds to the official definitions in the medical literature of between one and six standard drinks per week [9]. The information and counselling practice of health professionals and gynaecologists was described from the subjective point of view of the interviewees. A generalisation of the statements to all professionals is therefore not possible. Not all the stages identified here can be traced in every interview. In addition, in this paper, the perspective of the partners could only be included from the point of view of the women interviewed. Although a rough pattern of stages could be worked out, the applicability in each individual situation needs to be examined.

## Conclusion

In our study, we found that pregnant and breastfeeding women generally perceive alcohol consumption as a risk to the health of the child and would like to control this risk to a large extent. Adherence to the abstinence rule is particularly sought during pregnancy which, however, does not necessarily exclude occasional and low level consumption from the point of view of some women. Based on this perception, a range between occasional alcohol consumption up to the zero-tolerance limit is described as an acceptable way of dealing with the risk from the mothers’ point of view.

Many women express a need for guidance and counselling by health care professionals at some stages of the transition to motherhood. The stages identified can be used as pointers to address the subject of alcohol consumption during pregnancy and breastfeeding in real life settings. The long-term perspective offers insights into women’s changing needs during the transition from pregnancy to breastfeeding. The stage of pregnancy that has not yet been (medically) confirmed should be taken more into account, as women experience themselves as particularly vulnerable during this time. This also applies to the early stage of pregnancy until the epidemiological and perceived risks of miscarriage decreases. The results of the study therefore suggest that individual and low-threshold counselling services should be offered to women with low to moderate alcohol consumption. If possible, family centred counselling should take place before or in the stage around conception and be continued within the framework of continuous care models until the end of the breastfeeding period.

## Data Availability

Data is available on reasonable request by the corresponding author Jessica Pehlke-Milde.
